# Distinct systemic microbiome and microbial translocation are associated with plasma level of anti-CD4 autoantibody in HIV infection

**DOI:** 10.1038/s41598-018-31116-y

**Published:** 2018-08-27

**Authors:** Wanli Xu, Zhenwu Luo, Alexander V. Alekseyenko, Lisa Martin, Zhuang Wan, Binhua Ling, Zhiqiang Qin, Sonya L. Heath, Kendra Maas, Xiaomei Cong, Wei Jiang

**Affiliations:** 1 University of Connecticut School of Nursing, Storrs, Connecticut 06269 USA; 2 Department of Microbiology and Immunology, Medical University of South Carolina, Charleston, SC 29425 USA; 3 Program for Human Microbiome Research, Biomedical Informatics Center, Department of Public Health Sciences, Department of Oral Health Sciences, Medical University of South Carolina, Charleston, SC 29425 USA; 4 Division of Infectious Diseases, Department of Medicine, Medical University of South Carolina, Charleston, SC 29425 USA; 5 Department of Microbiology and Immunology, Tulane University School of Medicine, New Orleans, LA 70112 USA; 6 Tulane National Primate Research Center, New Orleans, LA 70433 USA; 7 Departments of Genetics, Louisiana State University Health Sciences Center, Louisiana Cancer Research Center, 1700 Tulane Ave., New Orleans, LA 70112 USA; 8 Division of Infectious Diseases, Department of Medicine, University of Alabama at Birmingham, Birmingham, AL 35294 USA; 9 Microbial Analysis, Resources, and Services, University of Connecticut, Storrs, CT 06269 USA

## Abstract

Microbial signals have been linked to autoantibody induction. Recently, we found that purified anti-CD4 autoantibodies from the plasma of chronic HIV-1-infected patients under viral-suppressed antiretroviral therapy (ART) play a pathologic role in poor CD4+ T cell recovery. The purpose of the study was to investigate the association of systemic microbiome and anti-CD4 autoantibody production in HIV. Plasma microbiome from 12 healthy controls and 22 HIV-infected subjects under viral-suppressed ART were analyzed by MiSeq sequencing. Plasma level of autoantibodies and microbial translocation (LPS, total bacterial 16S rDNA, soluble CD14, and LPS binding protein) were analyzed by ELISA, limulus amebocyte assay, and qPCR. We found that plasma level of anti-CD4 IgGs but not anti-CD8 IgGs was increased in HIV+ subjects compared to healthy controls. HIV+ subjects with plasma anti-CD4 IgG > 50 ng/mL (high) had reduced microbial diversity compared to HIV+ subjects with anti-CD4 IgG ≤ 50 ng/mL (low). Moreover, plasma anti-CD4 IgG level was associated with elevated microbial translocation and reduced microbial diversity in HIV+ subjects. The *Alphaproteobacteria* class was significantly enriched in HIV+ subjects with low anti-CD4 IgG compared to patients with high anti-CD4 IgG even after controlling for false discovery rate (FDR). The microbial components were different from the phylum to genus level in HIV+ subjects with high anti-CD4 IgGs compared to the other two groups, but these differences were not significant after controlling for FDR. These results suggest that systemic microbial translocation and microbiome may associate with anti-CD4 autoantibody production in ART-treated HIV disease.

## Introduction

Chronic inflammation or immune dysfunction has been a critical issue in human immunodeficiency virus (HIV) disease even in patients under viral suppressive antiretroviral therapy (ART). ART significantly suppresses HIV viral replication, improves immune function, and decreases morbidity and mortality in HIV disease^1,2^. However, a substantial number of patients fail to reconstitute their peripheral CD4+ T cell counts even after long-term viral-suppressive ART treatment, and exhibit increased risks of complications, morbidity and mortality^3–7^. Previous studies have shown that thymic and lymphatic fibrosis, low nadir CD4+ T cell counts, sustained increases in inflammation, and microbial translocation may account for patients with poor CD4+ T cell recovery under viral suppressive ART treatment^5,8–21^. However, the exact mechanism governing poor CD4+ T cell recovery is still unknown. In our recent work, we studied the anti-CD4 autoreactive IgGs purified from plasma of ART-treated aviremic patients with peripheral CD4+ T cell counts less than 350 cells/µL. Our study has shown that anti-CD4 autoreactive IgGs induce CD4+ T cell death through antibody-mediated natural killer (NK) cell cytotoxicity *in vitro*, suggesting that anti-CD4 autoantibodies play a role in blunted CD4+ T cell reconstitution after ART treatment^22^. Consistently, we have found that purified NK cells from patients with blunted CD4+ T cell recovery were enriched in cytotoxic cells and were able to mediate uninfected CD4+ T cell death *ex vivo*^23^.

Prior to ART treatment, HIV infection results in significant B cell depletion, especially memory B cell depletion, B cell hyperactivation and heightened plasma levels of autoantibodies, as well as impaired vaccine responsiveness^24–28^. These B cell perturbations cannot be completely explained by the lack of contribution from CD4+ T cells; B cell intrinsic defects have been observed^29,30^. For example, our previous work has shown that purified B cells from HIV-infected subjects had reduced proliferation capacities in response to toll-like receptor (TLR) 9 ligand stimulation compared to B cells from healthy controls *in vitro*^30^. Another study from Moir’s group reported that purified B cells from HIV-infected patients had reduced antigen-presenting function compared to B cells from healthy controls when co-culturing with purified T cells from the same healthy donors^29^. These results suggest B cell intrinsic dysfunction in HIV disease. Furthermore, B cells have been reported activated even after long-term viral-suppressive ART treatment, which may account for inconsistent serologic antibody responses and cellular responses in patients given seasonal influenza vaccination^31^.

The underlying mechanisms of long-term humoral immune perturbations in HIV-infected patients, despite undergoing ART treatment, are still largely unknown. The fecal microbiota and microbial translocation from the gastrointestinal (GI) tract to systemic circulation have been recently investigated as a major driver of immune perturbations and persistent systemic inflammation in HIV disease^32–35^. Increased intestinal permeability due to mucosal barrier dysfunction, GI immune dysregulation and/or altered intestinal microbiome are considered to be significant factors related to microbial translocation and HIV pathogenesis. Differences in fecal microbiome in HIV-infected patients versus healthy controls are associated with systemic inflammation^32^. Mechanistically, microbial products such as TLR ligands can induce autoantibody production and may play a pathogenic role in autoimmune diseases^36–38^. Increased systemic microbial translocation and its associated inflammation may result in B cell hyperactivation and perturbation in HIV disease. After long-term repeated stimulation by low concentrations of TLR ligands (compared to one dose and high concentration as vaccine adjuvants) and other microbial products released from the gut^24–26,39^, B cells may be polyclonally activated as reflected by increased total IgM and IgG^26,40^.

In the current study, we hypothesize that microbial translocation of specific bacterial strains may play a role in B cell activation and anti-CD4 autoantibody production. We, therefore, investigate systemic bacterial microbiome, the magnitude of microbial translocation, and plasma anti-CD4 autoantibodies in HIV+ subjects under long-term viral suppressive ART treatment.

## Methods

### Study Design, Subjects, and Data Collection

This study was approved by the Institutional Review Board at Medical University of South Carolina. All methods were performed in accordance with the relevant guidelines and regulations. All participants provided written informed consents. In the present study, 12 healthy volunteers and 22 HIV+ ART-treated aviremic (plasma HIV RNA < 50 copies/mL) patients were enrolled. The clinical characteristics of participants are shown in Table 1.

**Table 1:** Demographic and clinical characteristics of the participants.

	Healthy control	HIV+/**α**CD4^low^	HIV+/**α**CD4^high^	P1	P2	P3
Number	12	13	9			
Age	43.5 (33.5–56)	43 (26–46.5)	47 (36–56.5)	0.25	0.77	0.21
Gender (Male/%)	3 (25%)	11 (84.6%)	3 (33.3%)	0.005	>0.99	0.04
Race (AA/%)	7 (44%)	8 (57%)	7 (58%)	0.72	0.7	0.52
Nadir CD4 count (cells/*µ*L)		361 (226–490)	229 (124–426)			0.19
Duration of ART (yr)		4 (3.5–6.5)	6 (4–6)			0.82
CD4 count (cells/*µ*L)	828 (523–1043)	634 (514–744)	450 (321–677)	0.50	0.07	0.07
%ki67+ CD4	1.0 (0.7–1.6)	2.8 (1.9–3.8)	2.5 (1.7–3.9)	<0.0001	0.001	0.73
%annexin V+ CD4	19 (13.5–37.7)	29.4 (27.1–43)	26.9 (15.7–32)	0.14	0.60	0.18
B cell count (cells/*µ*L)	219 (112–235)	239 (132–314)	185 (130–245)	0.29	0.65	0.37
%ki67+ B cells	0.9 (0.7–1.1)	1.5 (0.9–2.0)	1.3 (0.9–2.7)	0.03	0.03	0.84
%annexin V+ B cells	9 (5.5–18)	19.4 (12.6–27.8)	17.6 (13.3–33)	0.007	0.04	0.86
Plasma soluble CD4 (ng/mL)	2.1 (1.2–4.9)	1.7 (0–2.7)	1.8 (0.3–2.7)	0.37	0.39	0.66
Current ART regimen						
Multi-Class Combination		11 (84.6%)	4 (44.4%)			>0.99
NRTIs		2 (15.4%)	3 (33.3%)			0.71
PIs		3 (23%)	3 (33.3%)			>0.99
Metabolic abnormities						
BMI		26.1 (23.3–29.7)	32.3 (24.9–38.3)			0.14
Diabetes mellitus		1 (0.08%)	1 (0.11%)			>0.99
Hypertension		4 (30.8%)	2 (22.2%)			0.67
Abnormal lipid metabolism		5 (38.5%)	3 (33.3%)			0.67

P1: HIV- vs HIV+/**α**CD4^low^.

P2: HIV- vs HIV+/**α**CD4^high^.

P3: HIV+/**α**CD4^high^ vs HIV+/**α**CD4^low^.

Non-parametric Mann-Whitney tests.

Abnormal lipid metabolism: hyperlipidemia, hyperlipidemia, hypertriglyceridemia, hypercholesterolemia.

Multi-Class Combination ART: Two different groups in a complete HIV drug regimen (e.g., Atripla (bictegravir + tenofovir DF + emtricitabine)).

### Inclusion and exclusion criteria

All participants were age 18 years and older. All patients had documented HIV infection and were receiving a stable antiretroviral regimen with plasma HIV RNA < 50 copies/mL more than two years prior to study entry. Transient viremic blips did not exclude participation if flanked by viral levels below detection limits. Exclusion criteria included pregnancy, breast-feeding, surgery, chemotherapy, inflammatory bowel diseases, and uses of steroids more than 10 mg per day for more than 120 days or uses of antibiotics within 14 days prior to enrollment.

### ELISA for detection of anti-CD4 IgGs and anti-CD8 IgGs

Human soluble CD4 protein (sCD4, Progenics Tarrytown, NY) or human soluble CD8B/P37/LEU2 protein (sCD8, Sino Biological Inc. Beijing, China) were diluted at the concentration of 16 μg/ml and added to microtiter wells, and incubated at 4 °C overnight. Microwells were washed three times with phosphate buffered saline wash buffer (PBS with 0.1% Tween 20), and then blocked with PBS containing 3% bovine serum albumin (BSA) for 120 min at 37 °C. Plasma was diluted 1:40 in PBS containing 3% BSA and 100 μl of the dilution were added to the wells. The plate was incubated at room temperature for 60 min. Biotin-labeled goat anti-human IgG was added at 1:5000 dilution in PBS containing 3% BSA. The plate was then incubated for 60 min at room temperature. Horseradish peroxidase conjugated streptavidin (HRP-Streptavidin) was added at a 1:1000 dilution in PBS containing 3% BSA, and then incubated for 30 min at room temperature. After washing, 100 μl 2,2′-Azino-di (3-ethylbenzthiazoline-6-sulfonate) were added and incubated for 30 min, and 405 nm emission was read within 30 min. PBS containing 3% BSA alone was used as a negative control and anti-CD4 and anti-CD8 antibodies were used as positive controls.

The 40th percentile (50 ng/mL) of anti-CD4 IgG was used to define the cutoff for high and low levels of the IgG. Therefore, patients with plasma anti-CD4 IgG level above 50 mg/mL were defined as the high anti-CD4 IgG group; and patients with plasma anti-CD4 IgG level equal or below 50 ng/mL were defined as the low anti-CD4 IgG group.

### Plasma levels of LPS, soluble CD14 (sCD14), LPS binding protein (LBP)

Plasma samples were collected into tubes containing EDTA and stored at −80 °C until they were thawed once. The method was described in our previous studies^41–43^. Briefly, the plasma samples were diluted to 10% with endotoxin-free water, and LPS was quantified using a commercially available limulus amebocyte assay kit (Lonza Inc., Allendale, NJ) according to the manufacturer’s protocol. sCD14 and LBP were quantified using kits from R&D (Minneapolis, MN) and Hycult Biotech (Plymouth Meeting, PA) respectively following manufacturers’ protocols.

### Quantitative polymerase chain reaction (PCR) for measurement of bacterial 16S rDNA

DNA was extracted from 400 μL endotoxin-free water and 400 μL plasma using QIAamp UCP pathogen Mini kit (Qiagen, Valencia, CA) according to the manufacturer’s instructions. The method was described in our previous studies^31,41^. Briefly, a 20 μL amplification reaction consisted of 10 μL of 2x Perfecta qPCR ToughMix (Quanta, Gaithersburg, MD), 0.3 μmol/L forward and reverse primers, 0.175 μmol/L probe (338 P: 5′-FAM-GCTGCCTCCCGTAGGAGT-BHQ1-3′), and 5 μL of the template plasma DNA. Degenerate forward (8 F: 5′-AGTTTGATCCTGGCTCAG-3′) and reverse (515 R: 5′-GWATTACCGCGGCKGCTG-3′) primers were used to amplify DNA templates encoding 16S rRNA. The DNA was amplified in duplicate, and mean values were calculated by subtracting values in the water control. A standard curve was created from serial dilutions of plasmid DNA containing known copy numbers of the template. The reaction conditions for amplification of DNA were 95 °C for 5 min, followed by 40 cycles at 95 °C for 15 s and at 60 °C for 1 min^41^.

### Plasma microbial DNA extraction, sequencing and data process

Microbial DNA extraction was described above in 16S rDNA assay. The 16S rRNA gene V4 variable region PCR primers 515/806 with barcode on the forward primer were used in a 30 cycle PCR (5 cycle used on PCR products) using the HotStarTaq Plus Master Mix Kit (Qiagen, USA) under the following conditions: 94 °C for 3 minutes, followed by 28 cycles of 94 °C for 30 seconds, 53 °C for 40 seconds and 72 °C for 1 minute, after which a final elongation step at 72 °C for 5 minutes was performed. After amplification, PCR products were checked in 2% agarose gel to determine the success of amplification and the relative intensity of bands. Multiple samples were pooled together (e.g., 100 samples) in equal proportions based on their molecular weight and DNA concentrations. Pooled samples were purified using calibrated Ampure XP beads. Then the pooled and purified PCR product was used to prepare the DNA library by following Illumina TruSeq DNA library preparation protocol. Sequencing was performed at MR DNA (www.mrdnalab.com, Shallowater, TX, USA) on a MiSeq following the manufacturer’s guidelines.

The Q25 sequence data derived from the sequencing process was processed using a proprietary analysis pipeline (www.mrdnalab.com, MR DNA, Shallowater, TX). Sequences were depleted of barcodes and primers, then short sequences <200 bp and sequences with ambiguous base calls, and sequences with homopolymer runs exceeding 6 bp were removed. Next, sequences were denoised and operational taxonomic units (OTUs) were defined clustering at 3% divergence (97% similarity) followed by removal of singleton sequences and chimeras^44–48^. Final OTUs were taxonomically classified using BLASTn against a curated database derived from GreenGenes, RDPII and NCBI^49^. The data has been summarized at each taxonomic level by both raw counts and relative abundances. For each plasma sample and water control, absolute and relative abundance in OTU tables were generated. To control for contamination, two water samples were used as negative controls for DNA extraction. ß-diversity is different from the samples of patients, healthy and water controls (Supplemental Fig. 1). In the data analysis, we used both methods of subtracting the mean abundance of the OTUs and removing any OTUs that are present in the water control. The PERMANOVA variability from both methods are the same. The results in this paper were presented based on the method of removing the mean absolute abundance of OTUs. See Supplemental Table 1 for the raw data of the read counts and relative abundance from each sample including water controls.

### Statistical Analysis

In the pre-specified hypothesis, we were interested in the comparisons of HIV+ high anti-CD4 antibody group versus HIV+ low anti-CD4 antibody group or healthy controls; therefore, P values from comparing HIV+ high anti-CD4 antibody group to each control group were not adjusted for multiple comparisons^50^. Non-parametric Mann-Whitney U tests were applied to the current study.

For microbiome analysis, OTU tables and different levels of taxonomy tables derived from the sequencing process described above were imported to R (version 3.3.1) for statistical analysis^51^. The mean values of two negative controls were subtracted from each sample’s OTU to control for the contamination. Simpson index of diversity was calculated using Vegan package^52^ to measure α diversity of each sample. Spearman’s Correlation test was used to assess the association among Simpson diversity index, clinical and demographic characters and autoreactive antibody. Bray-Curtis and Jaccard dissimilarity were calculated using Vegan package to evaluate ß-diversity, the compositional dissimilarity among the microbial community. Jaccard dissimilarity measures the dissimilarity between samples based on the presence/absence of the data, whereas Bray-Curtis dissimilarity was calculated based on both presence/absence and abundance. The relationships between ß-diversity of the microbial community and autoreactive antibody titer were assessed using PERMANOVA in Vegan package. Analysis of indicator species (Indicspecis package) was used to assess the relationship between the occurrence/abundance of species at the genus level with different clinical characters. False discovery rate (FDR) correction was applied to control for multiple comparisons.

### Accession codes

The data are available at the NCBI Sequence Read Archive (SRA) under accession no. SRP120355 (http://www.ncbi.nlm.nih.gov/sra).

## Results

A total of 34 participants completed the study, including 22 HIV patients and 12 healthy controls. Demographic characteristics of the participants are illustrated in Table 1.

### Plasma anti-CD4 IgG level but not anti-CD8 IgG level was increased in aviremic ART-treated HIV+ subjects compared to healthy controls

**Figure 1:**
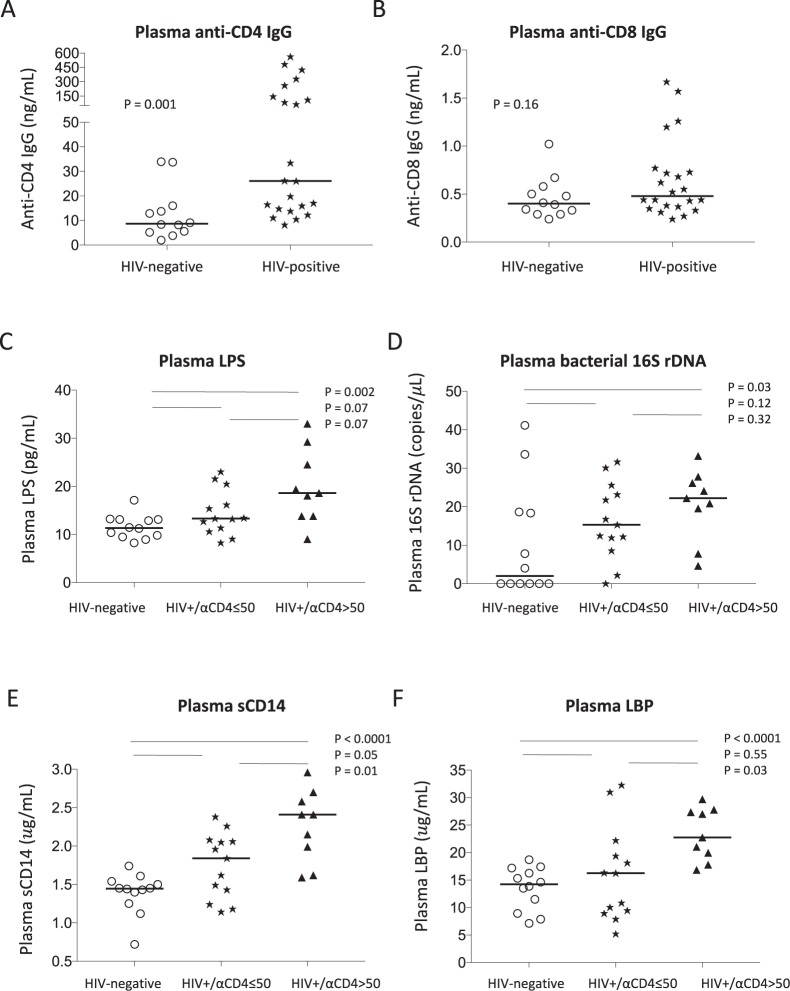
Plasma level of anti-CD4 IgG and its association with microbial translocation in HIV+ subjects. sCD4 and sCD8 proteins were used to detect plasma anti-CD4 IgGs (**A**) and anti-CD8 IgGs (**B**) by ELISA. Plasma levels of LPS were detected by limulus amebocyte assay (**C**), bacterial 16S rDNA were detected by qPCR (**D**), sCD14 (**E**) and LBP (**F**) by ELISA in healthy controls and HIV+ subjects with plasma anti-CD4 IgG > 50 ng/mL and ≤50 ng/mL. Non-parametric Mann-Whitney tests.

Following our recent work, we investigated the mechanism of anti-CD4 autoantibody production in well-controlled ART-treated HIV infection. We first analyzed plasma levels of anti-CD4 IgG as well as anti-CD8 IgG in age-matched healthy controls and aviremic ART-treated HIV-infected subjects. We found that the plasma level of autoreactive anti-CD8 IgG was similar in controls and HIV+ subjects, but the level of anti-CD4 IgG increased in the HIV+ subjects compared to controls (Fig. 1A,B), suggesting that B cell function is still abnormal even after long-term ART treatment and successful viral suppression.

### Plasma microbial translocation was elevated in HIV+ subjects with high plasma anti-CD4 IgGs compared to healthy controls

**Figure 2:**
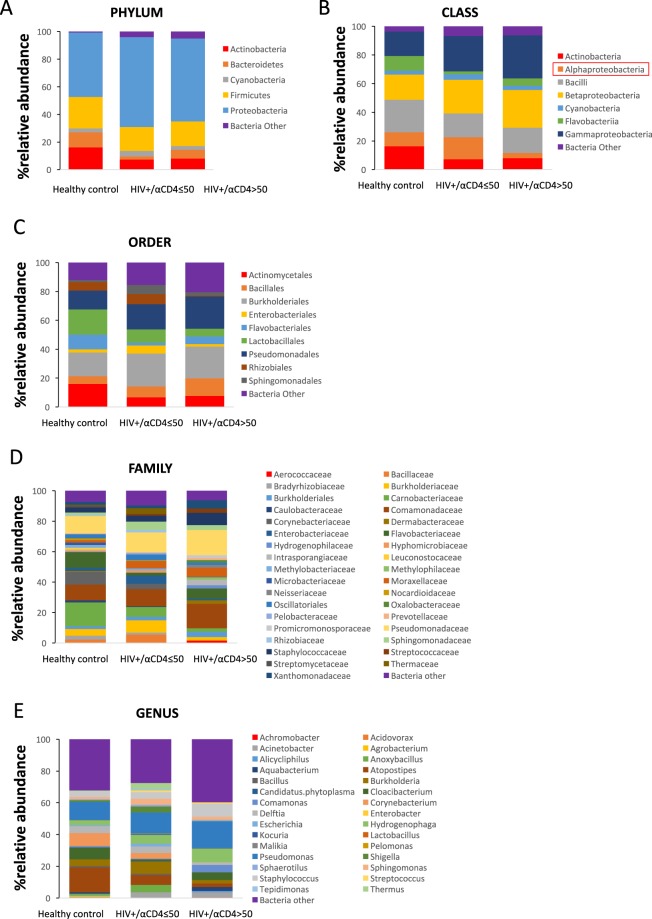
Circulating microbiome relative abundance analysis in healthy controls and HIV+ subjects. Microbial DNA was extracted from plasma and V4 variable region of bacterial 16S rDNA gene was amplified. The relative abundance of phylum (**A**), class (**B**), order (**C**), family (**D**), and genus (**E**) level bacteria (>1%) were shown in plasma from healthy controls, HIV+ subjects with plasma anti-CD4 IgG level ≤ 50 ng/mL and HIV+ subjects with anti-CD4 IgG > 50 ng/mL. The plasma enrichment of *Alphaproteobacteria* class was significantly higher in the low anti-CD4 IgG patient group compared to the high anti-CD4 IgG patient group after controlling for FDR.

Next, to investigate the association of systemic microbial translocation and plasma anti-CD4 IgGs level in HIV-infected subjects, we stratified patients to either high plasma autoantibody level or low plasma autoantibody level group. The cutoff value of 50 ng/mL plasma anti-CD4 IgG was defined based on 40 up-percentile, and no healthy controls were above that value. Notably, both plasma LPS level and bacterial 16S rDNA level, markers of microbial translocation^41^, tended to increase in HIV+ subjects with plasma anti-CD4 IgG below 50 ng/mL compared to healthy controls but have not achieved significant differences (Fig. 1C,D). Importantly, HIV+ subjects with high plasma level of anti-CD4 IgGs exhibited significantly elevated plasma microbial translocation (Fig. 2), suggesting that residual increased systemic microbial products may be associated with autoantibody production. In addition, we have evaluated the other two markers related to microbial translocation, sCD14 and LBP in plasma. Indeed, HIV+ subjects with high anti-CD4 IgGs had increased plasma sCD14 (Fig. 1E) and LBP (Fig. 1F) levels compared to the other two study groups. These results suggest that HIV+ subjects with high plasma anti-CD4 IgGs, but not HIV+ subjects with low plasma anti-CD4 IgGs, had increased systemic microbial translocation compared to healthy controls.

### Distinct plasma microbial profiles in HIV+ subjects with high anti-CD4 IgGs compared to controls

To investigate the difference of microbial translocation in healthy controls and HIV+ subjects, we performed and analyzed plasma microbiome (Fig. 2A–E). The samples yielded a total of 1,218,338 reads with an average of 34758.15 (±15380.71) reads per subject and 18280.5 (±10127.89) reads for water control. A total of 2408 OTUs were found in samples of all 34 subjects. On average, 400 (±98) OTUs were found in each sample. In contrast, 439 OTUs (average 272 ± 76) were found in the water control, and the top phyla were *Proteobacteria (*79.3%), *Firmicutes (*12.5%), *Deinococcus-Thermus (*7.3%), *Cuampbacteroa* (0.7%) and *Actinobacteria* (0.2%). In the phylum levels among all samples, 57.4% were *Proteobacteria*, 19.2% were *Firmicutes*, 10.5% were *Actinobacteria*, and 6.4% *Bacteroidetes* in plasma (Fig. 2A). A decreased ratio of *Firmicutes/Bacteroidetes* was reported on the fecal microbiome in autoantibody-derived autoimmune disease such as systemic lupus erythematosus (SLE)^53,54^. In this study, the ratios of *Firmicutes/Bacteroidetes* were 0.58 ± 0.45 in healthy controls, 0.37 ± 0.38 in the low anti-CD4 IgG HIV+ subjects, and 0.32 ± 0.30 in the high anti-CD4 IgG HIV+ subjects, respectively, but did not achieve significant difference between any two groups (mean ± SD, P > 0.05). At the class level, *Gammaproteobateria, Betaproteobacteria, Bacilli* and *Alphaproteobacteria* were predominant (80.3%) in the low anti-CD4 IgG group (Fig. 2B). Notably, the plasma enrichment of *Alphaproteobacteria* class was significantly higher in the low anti-CD4 IgG patient group compared to the high anti-CD4 IgG patient group after controlling for FDR (t = 3.22, P < 0.05, Fig. 2B). At the family level, *Staphylococcaceae* and *Pseudomonadaceae* were increased in the high anti-CD4 IgG patient group compared to the other two groups (Fig. 2D). At the genus level, *Alicycliphilus, Pseudomonas*, and *Staphylococcus* had increased relative abundance in the high anti-CD4 IgG patient group compared to the low anti-CD4 IgG patient group. (Fig. 2E). Although the microbial components were different from the phylum to genus levels in HIV+ subjects with high anti-CD4 IgGs compared to the other two groups, these differences were not significant after controlling for FDR.

### Reduced plasma microbial diversity was associated with increased plasma anti-CD4 antibodies in HIV-infected individuals

**Figure 3:**
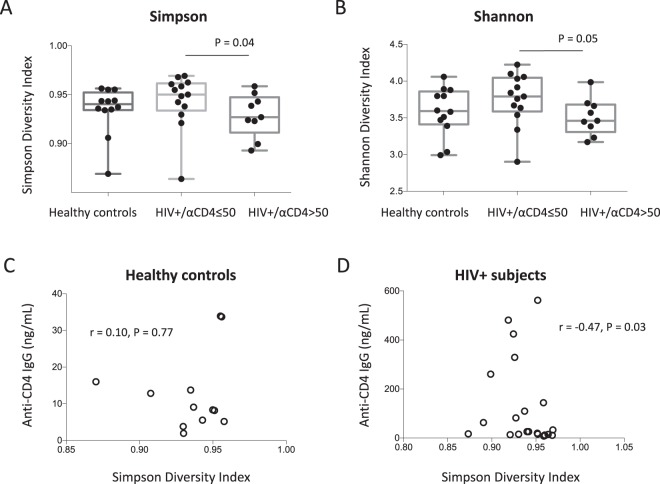
Reduced diversity was associated with increased plasma level of anti-CD4 autoantibody in HIV+ subjects. Box and whiskers plots of the Simpson (**A**) and Shannon (**B**) diversity indexes of plasma samples from HIV+ subjects with anti-CD4 IgG levels ≤ 50 ng/mL, >50 ng/mL and healthy controls. The top and bottom boundaries of each box indicate the 3^rd^ and 1^st^ quartile values, respectively. The central horizontal line represents the median values. The dot represents Simpson and Shannon diversity index of each sample. Non-parametric Mann-Whitney U tests. Correlations between the Simpson diversity index and plasma anti-CD4 IgG levels in healthy controls (**C**) and HIV+ subjects (**D**). Spearman correlation tests.

**Figure 4:**
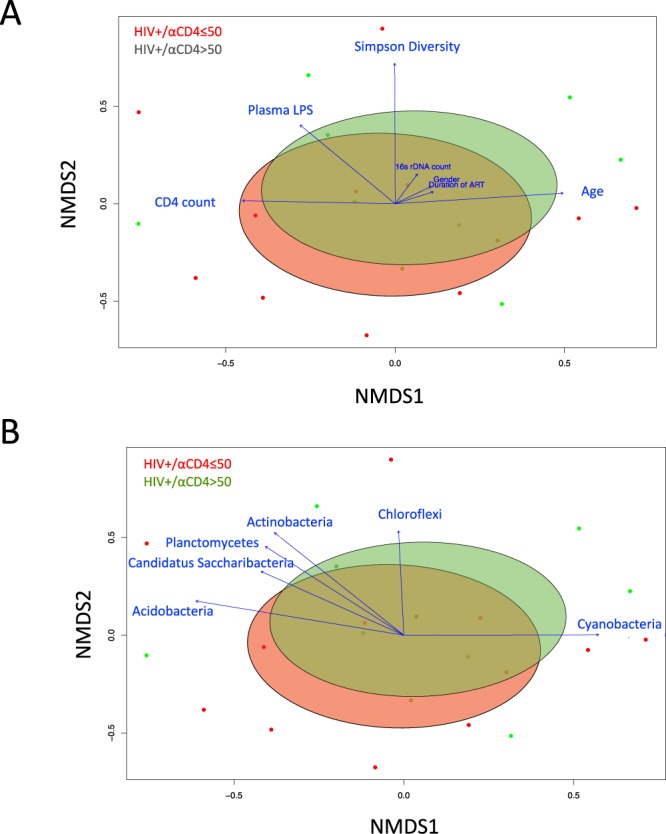
Nonmetric multidimensional scaling ordination (NMDS) plot of the OTUs with fitted vectors of clinical variables (**A**), and based on the abundance of bacterial phyla (**B**). Dots with different colors represent data from each plasma sample in HIV+ subjects with anti-IgG level ≤ 50 ng/mL (red) and HIV+ subjects with anti-CD4 IgG > 50 ng/mL (green). Ellipses denote the standard deviation of the weighted average NDMS score of anti-IgG level ≤ 50 ng/mL group (red) and anti-CD4 IgG > 50 ng/mL group (green). Community differences were verified by PERMONOVA test (Adonis, P < 0.05). Arrows represent the direction and magnitude of correlation of each clinical variable (**A**) and the abundance of bacterial phyla (**B**) with the ordination axes.

Next, to investigate the difference of composition in plasma microbiome in the three study groups, we analyzed microbial diversity including Simpson Diversity Index, Shannon index and species number observed. The Simpson and Shannon diversity indexes in the high anti-CD4 IgG HIV+ subject group were significantly lower compared to the low anti-CD4 IgG HIV+ subject group (P = 0.04 and P = 0.05 respectively, Fig. 3A,B). The numbers of species were 365.8 ± 99.6 in healthy controls, 373.5 ± 91.2 in the low anti-CD4 IgG HIV+ subjects, and 362.1 ± 85.7 in the high anti-CD4 IgG HIV+ subjects, respectively (mean ± SD, P > 0.05). There was an inverse correlation between plasma anti-CD4 IgG level and the Simpson diversity index in HIV+ subjects but not in healthy controls (Fig. 3C,D). Moreover, ß-diversity, the compositional dissimilarity among the microbial community was assessed using nonmetric dimensional scaling with both Bray-Curtis Coefficient and Jaccard Index, and revealed significant clusters between HIV-infected subjects with plasma anti-CD4 IgG level > 50 ng/mL and their counterparts (Fig. 4). Nonetheless, anti-CD4 IgG level explained 6.8% of the variation of Bray-Curtis coefficient among HIV-infected individuals after controlling for plasma LPS level, duration of the ART treatment and CD4 counts (PERMANOVA, n = 22, P < 0.05); PERANOVA test of anti-CD4 IgG level on Jaccard Index yielded a similar result. Indicator species analysis showed that patients who had a higher level of anti-CD4 IgG (>50 ng/mL) had significantly higher levels of *Alicycliphilus* (P < 0.05) and *Hylemonella* (P < 0.05). However, the significances disappeared after controlling for FDR.

## Discussion

Increased levels of autoreactive antibodies or autoimmune diseases have been shown in HIV/SIV infection^55–62^. ART treatment reduces B cell hyperactivation^63^. Our recent study shows that anti-CD4 autoantibodies purified from plasma of immunologic non-responders (undetectable plasma viral load, ART-treated, and CD4+ T cell counts <350 cells/µL) mediated CD4+ T cell death through antibody-dependent NK cell cytotoxicity, suggesting that anti-CD4 IgG plays a role in poor CD4+ T cell recovery under viral suppressive ART treatment^22^. In the current study, we found that both quantity and quality of plasma microbial products in ART-treated HIV-infected subjects was associated with anti-CD4 autoantibodies.

Microbial TLR and its agonists play a role in autoantibody production and autoimmune diseases^64,65^. Our previous study showed that plasma level of TLR4 ligand LPS was associated with inflammation and B cell activation in HIV disease^43^. Although ART treatment greatly reduces cell apoptosis and activation and thus limits autoantibody production^43,66–69^, we found that anti-CD4 specific antibody is a key exception (Fig. 1A). Moreover, altered B cell receptor (BCR) and TLR signals (e.g., MyD88) may promote autoreactive B cell selection^70^. Indeed, HIV+ subjects had elevated levels of microbial translocation (Fig. 1C,D) and cycling B cells^31^ compared to healthy controls, implying that bacterial products (e.g., LPS) may play a role in activating B cells. Nonetheless, how microenvironmental and inflammatory factors drive the breakdown of B cell tolerance, especially in humans, is not fully understood. Notably, autoimmune diseases in HIV are often observed after ART^55,71,72^, implying that pathologic autoantibodies are developed post the ART treatment.

Interestingly, a diverse bacterial DNAs were found in the plasma of healthy controls (Fig. 2). These findings are consistent with the study from Païssé S^73^. Low levels of microbial translocation occur in healthy individuals but increase when there is a GI barrier disruption. On the other hand, dysbiosis of gut microbiome community may result in mucosal immune dysfunction and intestinal mucosal barrier damage, which allows gut microbial translocation to the bloodstream^74–78^. Increased “leakiness” of microbial products (e.g., LPS) from the intestinal barrier further may cause systemic immune cell activation and drives immune perturbations^32^. Interestingly, we observed a trend decrease in the *Firmicutes/Bacteroidetes* ratio in HIV+ subjects with high anti-CD4 IgG level compared to the other two groups, which is consistent with prior reports on the fecal microbiome in autoimmune disease such as systemic lupus erythematosus (SLE)^53,54^.

Most microbiome studies used stool, saliva, or cervical-vaginal lavage fluid samples, very rare study was done on plasma microbiome due to highly technical demands^32,79–81^. A recent study reported that HIV-infected patients had different fecal microbial community composition compared to healthy controls^32^. Fecal microbiome from HIV-infected patients was enriched in *Enterobacteriales, Erysipelotrichaceae, Proteobacteria, Enterobacteriaceae, Gammaproteobacteria, Erysipelotrichi, Barnesiella*, and *Erysipelotrichales*, but was depleted in *Rikenellaceae* and *Alistipes*, relative to healthy controls^32^. Another study showed that HIV-infected patients with low peripheral CD4+ T cell counts exhibited reduced enteric bacterial diversity, which is consistent with our findings^79^. Both studies indicate that enrichment of *Enterobacteriaceae* was associated with systemic inflammation^32,79^. Consistently, plasma enrichment of *Proteobacteria, Gammaproteobacteria* and *Betaproteobacteria* was also observed in HIV+ individuals compared to healthy controls in the current study, but the difference did not achieve statistical significance (Fig. 2). However, we did not observe enrichment in other bacteria products reported in the fecal microbiome study besides *Proteobacteria, Gammaproteobacteria* and *Betaproteobacteria* in plasma from HIV+ individuals relative to healthy controls^32^. Nonetheless, it is important to investigate microbiome simultaneously in plasma and mucosal sites in HIV in the future.

TLR4 signaling was increased with transgenic mice for a TLR chaperone molecule (gp96), which resulted in a lupus-like autoimmune glomerulonephritis^26^. Flares of autoimmune diseases have been observed with infection^82^ in humans and also is an inducer of autoimmunity in mice. Decreased anti-dsDNA antibodies were observed in TLR2 and TLR4 knockout C57BL/6 (lpr/lpr) mice; and autoantibodies were induced by LPS stimulation through the TLR4-dependent cell signaling pathway in lupus-prone mice^83,84^. Therefore, increased bacterial product translocation may play a key role to induce autoantibodies in HIV. However, the association of plasma bacterial products (e.g., LPS) and anti-CD4 IgG level we observed in the current study does not prove causality. Next, we will give HIV-infected humanized animal models with specific bacterial products (e.g., LPS) found in plasma of the high HIV+ subjects to evaluate anti-CD4 autoantibody production. The other possibility of this association can be high anti-CD4 autoantibody-mediated immunodeficiency (poor CD4+ T cell recovery^22^) and increased inflammation favor particular bacterial survival. Furthermore, plasma soluble CD4 level was similar among the three study groups^22^, suggesting that increased anti-CD4 IgG in some patients may not result from increased antigens in plasma. However, we do not know whether the level of CD4 antigen and HIV proteins (e.g., gp120^85^) with CD4 binding capacity is increased in lymph nodes, raising the question that increased anti-CD4 IgG may be due to increased antigens in the patients with high anti-CD4 IgG level.

Women in general have higher humoral and cellular immune responses relative to men, as well as higher prevalence of autoimmune diseases^86^. Mechanisms accounting for sex differences in autoimmune diseases include sex-induced breaks in tolerance and increases in peripheral cell activity, such as TLR responsiveness, T regulatory cells, environmental and genetic factors^87–90^. Consistently, we found that there were more women in the high HIV+ anti-CD4 autoantibody group compared to the low HIV+ anti-CD4 autoantibody group (Table 1). Whether anti-CD4 autoantibody induced by female sex hormones or sex hormone-mediated immune responses is worth further investigation.

This is the first study to date to report plasma microbiome and microbial products (e.g., LPS) in relation to autoantibodies in HIV patients. One of its limitations remains a small sample size. Due to the small sample size and large amount of microbial species observed in the plasma, most significant differences of microbiome among the study groups were not demonstrable after FDR correction. Another limitation is that other factors that may influence gut microbiota composition and bacterial translocation, such as diet, usage of probiotics and antibiotics, and the comorbidity of the patients were not controlled in the study. Therefore, the interpretation and generalization of findings may be limited. Future studies with large and diverse sample sizes are needed to lead a greater understanding of the concept of microbial translocation and auto-immune responses. In addition, the contributing factors for microbiome including sex should be considered.

In summary, we found that elevated plasma anti-CD4 IgG in HIV-infected subjects was associated with the magnitude of systemic microbial translocation and systemic microbiome. At the class level, *Gammaproteobateria, Betaproteobacteria, Bacilli* and *Alphaproteobacteria* were predominant in the low anti-CD4 IgG group. At the genus level, *Alicycliphilus*, and *Hylemonella* had elevated relative abundance in the high anti-CD4 IgG patient group compared to the low anti-CD4 IgG patient group. These results suggest that systemic microbial translocation and microbiome may play a role in anti-CD4 autoantibody production in HIV infection. However, the small sample size in the current study prevents us to draw further conclusions.

## Electronic supplementary material

Supplemental figure 1

Supplemental table 1

## References

[1] Keiser O (2004). All cause mortality in the Swiss HIV Cohort Study from 1990 to 2001 in comparison with the Swiss population. AIDS.

[2] Palella FJ (2006). Mortality in the highly active antiretroviral therapy era: changing causes of death and disease in the HIV outpatient study. J Acquir Immune Defic Syndr.

[3] Baker JV (2008). CD4+ count and risk of non-AIDS diseases following initial treatment for HIV infection. Aids.

[4] Lewden C (2007). HIV-infected adults with a CD4 cell count greater than 500 cells/mm3 on long-term combination antiretroviral therapy reach same mortality rates as the general population. J Acquir Immune Defic Syndr.

[5] Lederman MM (2011). Immunologic failure despite suppressive antiretroviral therapy is related to activation and turnover of memory CD4 cells. The Journal of infectious diseases.

[6] Kelley CF (2009). Incomplete peripheral CD4+ cell count restoration in HIV-infected patients receiving long-term antiretroviral treatment. Clinical infectious diseases: an official publication of the Infectious Diseases Society of America.

[7] Gutierrez F (2008). Patients’ characteristics and clinical implications of suboptimal CD4 T-cell gains after 1 year of successful antiretroviral therapy. Current HIV research.

[8] Moore RD, Keruly JC (2007). CD4+ cell count 6 years after commencement of highly active antiretroviral therapy in persons with sustained virologic suppression. Clinical infectious diseases: an official publication of the Infectious Diseases Society of America.

[9] Tuboi SH (2007). Discordant responses to potent antiretroviral treatment in previously naive HIV-1-infected adults initiating treatment in resource-constrained countries: the antiretroviral therapy in low-income countries (ART-LINC) collaboration. J Acquir Immune Defic Syndr.

[10] Hunt PW (2003). T cell activation is associated with lower CD4+ T cell gains in human immunodeficiency virus-infected patients with sustained viral suppression during antiretroviral therapy. The Journal of infectious diseases.

[11] Fernandez S, Price P, McKinnon EJ, Nolan RC, French MA (2006). Low CD4+ T-cell counts in HIV patients receiving effective antiretroviral therapy are associated with CD4+ T-cell activation and senescence but not with lower effector memory T-cell function. Clinical immunology.

[12] Benveniste O (2005). Mechanisms involved in the low-level regeneration of CD4+ cells in HIV-1-infected patients receiving highly active antiretroviral therapy who have prolonged undetectable plasma viral loads. J Infect Dis.

[13] Marchetti G (2006). Comparative analysis of T-cell turnover and homeostatic parameters in HIV-infected patients with discordant immune-virological responses to HAART. Aids.

[14] Marchetti G (2008). Microbial translocation is associated with sustained failure in CD4+ T-cell reconstitution in HIV-infected patients on long-term highly active antiretroviral therapy. Aids.

[15] Rajasuriar R (2010). Biological determinants of immune reconstitution in HIV-infected patients receiving antiretroviral therapy: the role of interleukin 7 and interleukin 7 receptor alpha and microbial translocation. J Infect Dis.

[16] Gandhi RT (2006). Effect of baseline- and treatment-related factors on immunologic recovery after initiation of antiretroviral therapy in HIV-1-positive subjects: results from ACTG 384. J Acquir Immune Defic Syndr.

[17] Anthony KB (2003). Incomplete CD4 T cell recovery in HIV-1 infection after 12 months of highly active antiretroviral therapy is associated with ongoing increased CD4 T cell activation and turnover. J Acquir Immune Defic Syndr.

[18] Valdez H (2002). Limited immune restoration after 3 years’ suppression of HIV-1 replication in patients with moderately advanced disease. AIDS.

[19] Negredo E (2010). Nadir CD4 T cell count as predictor and high CD4 T cell intrinsic apoptosis as final mechanism of poor CD4 T cell recovery in virologically suppressed HIV-infected patients: clinical implications. Clinical infectious diseases: an official publication of the Infectious Diseases Society of America.

[20] Teixeira L (2001). Poor CD4 T cell restoration after suppression of HIV-1 replication may reflect lower thymic function. Aids.

[21] Molina-Pinelo S (2009). Premature immunosenescence in HIV-infected patients on highly active antiretroviral therapy with low-level CD4 T cell repopulation. J Antimicrob Chemother.

[22] Luo Z. W. *et al*. Pathological role of anti-CD4 antibodies in HIV-infected immunologic non-responders under viral suppressive antiretroviral therapy. *Journal of Infectious Diseases* Epub ahead of print, 10.1093/infdis/jix223 (2017). 10.1093/infdis/jix223 PMC5853506 28498953

[23] Luo Z (2017). Increased Natural Killer Cell Activation in HIV-Infected Immunologic Non-Responders Correlates with CD4+ T Cell Recovery after Antiretroviral Therapy and Viral Suppression. PLoS One.

[24] Titanji K (2006). Loss of memory B cells impairs maintenance of long-term serologic memory during HIV-1 infection. Blood.

[25] De Milito A, Morch C, Sonnerborg A, Chiodi F (2001). Loss of memory (CD27) B lymphocytes in HIV-1 infection. AIDS (London, England).

[26] De Milito A (2004). Mechanisms of hypergammaglobulinemia and impaired antigen-specific humoral immunity in HIV-1 infection. Blood.

[27] Titanji K (2005). Primary HIV-1 infection sets the stage for important B lymphocyte dysfunctions. AIDS (London, England).

[28] Guan Y (2009). Discordant memory B cell and circulating anti-Env antibody responses in HIV-1 infection. Proceedings of the National Academy of Sciences of the United States of America.

[29] Malaspina A (2003). Deleterious effect of HIV-1 plasma viremia on B cell costimulatory function. J Immunol.

[30] Jiang W (2008). Impaired naive and memory B-cell responsiveness to TLR9 stimulation in human immunodeficiency virus infection. J Virol.

[31] Luo Z (2016). Key differences in B cell activation patterns and immune correlates among treated HIV-infected patients versus healthy controls following influenza vaccination. Vaccine.

[32] Dinh DM (2015). Intestinal microbiota, microbial translocation, and systemic inflammation in chronic HIV infection. J Infect Dis.

[33] Mutlu EA (2014). A compositional look at the human gastrointestinal microbiome and immune activation parameters in HIV infected subjects. PLoS Pathog.

[34] Nowak P (2015). Gut microbiota diversity predicts immune status in HIV-1 infection. AIDS.

[35] Villanueva-Millan MJ, Perez-Matute P, Recio-Fernandez E, Lezana Rosales JM, Oteo JA (2017). Differential effects of antiretrovirals on microbial translocation and gut microbiota composition of HIV-infected patients. J Int AIDS Soc.

[36] Umiker BR (2014). Dosage of X-linked Toll-like receptor 8 determines gender differences in the development of systemic lupus erythematosus. European journal of immunology.

[37] Wong CK (2010). Activation profile of Toll-like receptors of peripheral blood lymphocytes in patients with systemic lupus erythematosus. Clin Exp Immunol.

[38] Thibault DL (2008). IRF9 and STAT1 are required for IgG autoantibody production and B cell expression of TLR7 in mice. The Journal of clinical investigation.

[39] Brenchley JM (2006). Microbial translocation is a cause of systemic immune activation in chronic HIV infection. Nature medicine.

[40] Nagase H (2001). Mechanism of hypergammaglobulinemia by HIV infection: circulating memory B-cell reduction with plasmacytosis. Clinical immunology (Orlando, Fla.

[41] Jiang W (2009). Plasma levels of bacterial DNA correlate with immune activation and the magnitude of immune restoration in persons with antiretroviral-treated HIV infection. J Infect Dis.

[42] Jiang W (2014). Cycling memory CD4+ T cells in HIV disease have a diverse T cell receptor repertoire and a phenotype consistent with bystander activation. J Virol.

[43] Zhang L (2014). Plasmacytoid dendritic cells mediate synergistic effects of HIV and lipopolysaccharide on CD27+ IgD- memory B cell apoptosis. Journal of virology.

[44] Dowd SE (2008). Evaluation of the bacterial diversity in the feces of cattle using 16S rDNA bacterial tag-encoded FLX amplicon pyrosequencing (bTEFAP). BMC Microbiol.

[45] Dowd SE, Sun Y, Wolcott RD, Domingo A, Carroll JA (2008). Bacterial tag-encoded FLX amplicon pyrosequencing (bTEFAP) for microbiome studies: bacterial diversity in the ileum of newly weaned Salmonella-infected pigs. Foodborne Pathog Dis.

[46] Capone KA, Dowd SE, Stamatas GN, Nikolovski J (2011). Diversity of the human skin microbiome early in life. J Invest Dermatol.

[47] Eren AM (2011). Exploring the diversity of Gardnerella vaginalis in the genitourinary tract microbiota of monogamous couples through subtle nucleotide variation. PLoS One.

[48] Swanson KS (2011). Phylogenetic and gene-centric metagenomics of the canine intestinal microbiome reveals similarities with humans and mice. ISME J.

[49] DeSantis TZ (2006). Greengenes, a chimera-checked 16S rRNA gene database and workbench compatible with ARB. Appl Environ Microbiol.

[50] Rothman KJ (1990). No adjustments are needed for multiple comparisons. Epidemiology.

[51] Team, R. C. *R: A language and environment for statistical computing*. *R Foundation for Statistical Computing* (2016).

[52] Jari Oksanen, F. G. B. *et al*. Eduard Szoecs and Helene Wagner vegan: Community Ecology Package. *R package version* 2.4–1. doi:CRAN.R-project.org/package=vegan (2016).

[53] Hevia A (2014). Intestinal Dysbiosis Associated with Systemic Lupus Erythematosus. mBio.

[54] He Z, Shao T, Li H, Xie Z, Wen C (2016). Alterations of the gut microbiome in Chinese patients with systemic lupus erythematosus. Gut pathogens.

[55] Sheikh V (2014). Graves’ disease as immune reconstitution disease in HIV-positive patients is associated with naive and primary thymic emigrant CD4(+) T-cell recovery. AIDS.

[56] Shah I (2013). Immune thrombocytopenic purpura: a presentation of HIV infection. Journal of the International Association of Providers of AIDS Care.

[57] Visser R, de Mast Q, Netea-Maier RT, van der Ven AJ (2012). Hashimoto’s thyroiditis presenting as acute painful thyroiditis and as a manifestation of an immune reconstitution inflammatory syndrome in a human immunodeficiency virus-seropositive patient. Thyroid: official journal of the American Thyroid Association.

[58] Bonsignori M (2014). An autoreactive antibody from an SLE/HIV-1 individual broadly neutralizes HIV-1. The Journal of clinical investigation.

[59] Levesque MC (2009). Polyclonal B cell differentiation and loss of gastrointestinal tract germinal centers in the earliest stages of HIV-1 infection. PLoS medicine.

[60] Kuwata T (2009). Association of progressive CD4(+) T cell decline in SIV infection with the induction of autoreactive antibodies. PLoS pathogens.

[61] Stahl D (2005). Alterations of self-reactive antibody repertoires in HIV disease: an insight into the role of T cells in the selection of autoreactive B cells. Immunol Lett.

[62] Rodriguez-Mahou M (1994). Autoimmune phenomena in children with human immunodeficiency virus infection and acquired immunodeficiency syndrome. Acta paediatrica.

[63] Nilssen DE, Oktedalen O, Brandtzaeg P (2004). Intestinal B cell hyperactivity in AIDS is controlled by highly active antiretroviral therapy. Gut.

[64] Lartigue A (2009). Critical role of TLR2 and TLR4 in autoantibody production and glomerulonephritis in lpr mutation-induced mouse lupus. Journal of immunology.

[65] Simchoni N, Cunningham-Rundles C (2015). TLR7- and TLR9-Responsive Human B Cells Share Phenotypic and Genetic Characteristics. J Immunol.

[66] du Toit R, Whitelaw D, Taljaard JJ, du Plessis L, Esser M (2011). Lack of specificity of anticyclic citrullinated peptide antibodies in advanced human immunodeficiency virus infection. J Rheumatol.

[67] Fust G (2005). Antibodies against heat shock proteins and cholesterol in HIV infection. Mol Immunol.

[68] Pereda I (2001). Antitissue transglutaminase antibodies in HIV infection and effect of highly active antiretroviral therapy. J Acquir Immune Defic Syndr.

[69] Horvath A (2001). High level of anticholesterol antibodies (ACHA) in HIV patients. Normalization of serum ACHA concentration after introduction of HAART. Immunobiology.

[70] Kolhatkar NS (2015). Altered BCR and TLR signals promote enhanced positive selection of autoreactive transitional B cells in Wiskott-Aldrich syndrome. J Exp Med.

[71] Zandman-Goddard G, Shoenfeld Y (2002). HIV and autoimmunity. Autoimmun Rev.

[72] Iordache L (2014). Autoimmune diseases in HIV-infected patients: 52 cases and literature review. Autoimmun Rev.

[73] Paisse S (2016). Comprehensive description of blood microbiome from healthy donors assessed by 16S targeted metagenomic sequencing. Transfusion.

[74] Barlow GM, Yu A, Mathur R (2015). Role of the Gut Microbiome in Obesity and Diabetes Mellitus. Nutr Clin Pract.

[75] Mathur R, Barlow GM (2015). Obesity and the microbiome. Expert Rev Gastroenterol Hepatol.

[76] Chiriac MT, Mahapatro M, Neurath MF, Becker C (2017). The Microbiome in Visceral Medicine: Inflammatory Bowel Disease, Obesity and Beyond. Visc Med.

[77] Bischoff SC (2017). [The intestinal microbiome and metabolic diseases: From obesity to diabetes and nonalcoholic steatohepatitis]. Internist (Berl).

[78] Bouter KE, van Raalte DH, Groen AK, Nieuwdorp M (2017). Role of the Gut Microbiome in the Pathogenesis of Obesity and Obesity-Related Metabolic Dysfunction. Gastroenterology.

[79] Monaco CL (2016). Altered Virome and Bacterial Microbiome in Human Immunodeficiency Virus-Associated Acquired Immunodeficiency Syndrome. Cell host & microbe.

[80] Lelouvier B (2016). Changes in blood microbiota profiles associated with liver fibrosis in obese patients: A pilot analysis. Hepatology.

[81] Ericsen AJ (2016). Microbial Translocation and Inflammation Occur in Hyperacute Immunodeficiency Virus Infection and Compromise Host Control of Virus Replication. PLoS Pathog.

[82] Segal Y, Calabro M, Kanduc D, Shoenfeld Y (2017). Human papilloma virus and lupus: the virus, the vaccine and the disease. Current opinion in rheumatology.

[83] Qing X (2008). Pathogenic anti-DNA antibodies modulate gene expression in mesangial cells: involvement of HMGB1 in anti-DNA antibody-induced renal injury. Immunol Lett.

[84] Zhai JX (2012). PDTC attenuate LPS-induced kidney injury in systemic lupus erythematosus-prone MRL/lpr mice. Mol Biol Rep.

[85] Zhang, Y. *et al*. The glycan-mediated mechanism on the interactions of gp120 with CD4 and antibody: Insights from molecular dynamics simulation. *Chem Biol Drug Des*, 10.1111/cbdd.13045 (2017). 10.1111/cbdd.13045 28632942

[86] Alpizar-Rodriguez D, Pluchino N, Canny G, Gabay C, Finckh A (2017). The role of female hormonal factors in the development of rheumatoid arthritis. Rheumatology (Oxford).

[87] Afshan G, Afzal N, Qureshi S (2012). CD4+ CD25(hi) regulatory T cells in healthy males and females mediate gender difference in the prevalence of autoimmune diseases. Clinical laboratory.

[88] Pisitkun P (2006). Autoreactive B cell responses to RNA-related antigens due to TLR7 gene duplication. Science.

[89] Rullo OJ, Tsao BP (2013). Recent insights into the genetic basis of systemic lupus erythematosus. Annals of the rheumatic diseases.

[90] Brusca SB, Abramson SB, Scher JU (2014). Microbiome and mucosal inflammation as extra-articular triggers for rheumatoid arthritis and autoimmunity. Current opinion in rheumatology.

