# Supplementary material for: Distinct systemic microbiome and microbial translocation are associated with plasma level of anti-CD4 autoantibody in HIV infection
Source: Sci Rep. 2018 Aug 27;8:12863. doi: 10.1038/s41598-018-31116-y (PMC6110826; doi:10.1038/s41598-018-31116-y)
Supplement: Supplementary file 1 — Supplemental figure 1 [file 41598_2018_31116_MOESM1_ESM.docx]

Distinct systemic microbiome and microbial translocation are associated with plasma level of anti-CD4 autoantibody in HIV infection

Running title: Systemic microbiome and anti-CD4 IgGs in HIV

Wanli Xu^1*^, Zhenwu Luo^2*^, Alexander V. Alekseyenko^3^, Lisa Martin^4^, Zhuang Wan^2^, Binhua Ling^5^, Zhiqiang Qin^6^, Sonya Heath^7^, Kendra Maas^8^, Xiaomei Cong^1#^, Wei Jiang^2, 4#^

^1^University of Connecticut School of Nursing, Storrs, Connecticut, USA, 06269

^2^Department of Microbiology and Immunology, Medical University of South Carolina, Charleston, SC, USA, 29425

^3^Program for Human Microbiome Research, Biomedical Informatics Center, Department of Public Health Sciences, Department of Oral Health Sciences, Medical University of South Carolina, Charleston, SC, USA, 29425

^4^Division of Infectious Diseases, Department of Medicine, Medical University of South Carolina, Charleston, SC, USA, 29425

^5^Department of Microbiology and Immunology, Tulane University School of Medicine, New Orleans, LA, 70112; Tulane National Primate Research Center, New Orleans, LA, 70433

^6^Departments of Genetics, Louisiana State University Health Sciences Center, Louisiana Cancer Research Center, 1700 Tulane Ave., New Orleans, LA 70112, USA

^7^Division of Infectious Diseases, Department of Medicine, University of Alabama at Birmingham, Birmingham, AL, USA, 35294

^8^Microbial Analysis, Resources, and Services, University of Connecticut, Storrs, CT, USA, 06269

*The authors contribute the equal amount of work.

^#^Corresponding author: Wei Jiang, email: [jianw@musc.edu](mailto:jianw@musc.edu), mailing address: 173 Ashley Ave. BSB207, Charleston, SC, 29425; and Xiaomei Cong, email: [xiaomei.cong@uconn.edu](mailto:xiaomei.cong@uconn.edu)

**Supplemental figure 1.** Nonmetric multidimensional scaling ordination (NMDS) plot of the OTUs of plasma microbial samples from HIV+ subjects (blue), healthy controls (green), and water controls (red). Dots with different colors represent data from each sample. Ellipses denote the standard error of weighted average NDMS score of each group.
